# Ultra-low-dose cone-beam CT for cleft and craniofacial deformity surgery: radiation reduction and clinical applicability

**DOI:** 10.1186/s40902-026-00505-z

**Published:** 2026-03-04

**Authors:** Pilvi Mäntynen, Juha Koivisto, Junnu Leikola, Touko Kaasalainen, Noemi Sipos, Arja Heliövaara, Jani Horelli, Jan Wolff, Niilo Lusila, Juho Suojanen

**Affiliations:** 1 Faculty of Medicine, University of Helsinki, Helsinki, Finland; 2 Cleft palate and Craniofacial Centre, Department of Plastic Surgery, Helsinki University Hospital, Helsinki, Finland; 3 Department of Physics, University of Helsinki, Helsinki, Finland; 4 Planmeca Ltd, Helsinki, 00880 Finland; 5 Helsinki University Hospital, Helsinki, Finland; 6 Helsinki University and Helsinki University Hospital, HUS Diagnostic Center, Radiology, Helsinki, Finland; 7 Department of Oral and Maxillofacial Surgery, University Hospital Schleswig-Holstein, Kiel, Germany; 8 Deparetment of Radiology, Central Hospital, Päijät-Häme Joint Authority for Health and Wellbeing, Lahti, Finland; 9 Department of Oral and Maxillofacial Surgery, Central Hospital, Päijät-Häme Joint Authority for Health and Wellbeing, Lahti, Finland

**Keywords:** Ultra-low-dose CBCT, Cleft and dentofacial deformities, Virtual surgical planning, Patient-specific implant, Radiation reduction, Maxillofacial surgery

## Abstract

**Background:**

Patients with cleft lip and palate and other craniofacial deformities often require repeated imaging during growth, making radiation reduction a priority. Cone-beam computed tomography (CBCT) offers lower radiation exposure than conventional CT, and ultra-low-dose (ULD) protocols may further reduce dose while maintaining clinical usability for surgical planning.

**Objective:**

This retrospective cohort study evaluated whether ULD CBCT provides comparable clinical usability to standard-dose CBCT for virtual surgical planning (VSP) and patient-specific implant (PSI) design in children and young adults undergoing orthognathic surgery for pediatric-onset craniofacial conditions.

**Methods:**

Forty consecutive patients with pediatric-onset craniofacial anomalies (23 unilateral cleft lip and palate, 10 bilateral cleft lip and palate, and 7 other craniofacial deformities) who underwent PSI-based orthognathic surgery were included. The primary predictor was the CBCT protocol (standard-dose FACE vs. ULD JAW), selected in routine clinical practice according to institutional imaging protocols and case-specific assessment by the interpreting radiologist; no predefined allocation criteria were applied. Primary outcomes were intraoperative cutting guide fit and PSI fit, defined as stable anatomical seating without visible gap or rocking and no need for intraoperative modification. The secondary outcome was occlusal outcome (as planned/compromise/reoperated). Radiation exposure during the orthognathic surgical phase was quantified using dose–area product (DAP) and effective dose. Baseline comparability between protocol groups was explored using bivariate analyses and logistic regression.

**Results:**

The ULD protocol resulted in a significantly lower effective dose (40.3 µSv) compared with the standard-dose protocol (89.0 µSv, *p* < 0.001). No significant differences were observed in cutting guide fit (*p* = 0.450) or PSI fit (*p* = 0.238). In logistic regression analysis, age was associated with protocol allocation (*p* = 0.014), whereas sex, diagnosis, and procedure type were not. Thirty-seven of 40 PSIs were clinically acceptable intraoperatively. Both CBCT protocols were clinically adequate for PSI-based orthognathic planning.

**Conclusion:**

ULD CBCT provides sufficient image quality and intraoperative usability for VSP in cleft and dentofacial deformity surgery while significantly reducing radiation exposure. These findings support adherence to the ALARA principle in patients requiring repeated imaging.

## Introduction

 Cone-beam computed tomography (CBCT) has become integral to preoperative maxillofacial assessment, providing high-resolution three-dimensional visualization with lower radiation exposure than conventional CT [1]. Ultra-low-dose (ULD) protocols have been developed to further minimize exposure [2]. Dose reduction may be achieved through beam filtration, optimized reconstruction settings, reduced tube current or pulsed exposure, and adjusted tube voltage while maintaining diagnostic adequacy [3, 4].

CBCT is widely used in CAD/CAM–based virtual surgical planning (VSP) for orthognathic procedures such as Le Fort I (LF1) and bimaxillary osteotomies. Accurate bone segmentation and STL model generation are essential for manufacturing patient-specific implants (PSIs) and cutting guides, which improve surgical precision and predictability [5]. However, optimal visual image quality does not necessarily guarantee sufficient STL model fidelity for implant adaptation [6].

Patients with cleft lip and palate and other congenital craniofacial deformities often undergo repeated imaging throughout growth, increasing cumulative radiation dose. Because children are more radiosensitive, strict adherence to the ALARA principle is essential.

The aim of this study was to compare two CBCT protocols—JAW (ULD) and FACE (standard dose)—for VSP and PSI design in LF1 and bimaxillary osteotomies in patients with cleft lip and palate or other congenital craniofacial anomalies. We evaluated radiation exposure, STL model adequacy, and intraoperative usability, and explored potential limitations related to ULD reconstruction algorithms [7].

## Methods

### Study design and setting

This retrospective cohort study was conducted at the Cleft Palate and Craniofacial Centre, HUSUKE (Helsinki University Hospital, Helsinki, Finland), the national referral centre for cleft lip and palate treatment in Finland. Data were collected between January 2021 and December 2023. The study protocol was approved by the Regional Board for Research (HUS3368).

### Eligibility criteria

Consecutive patients undergoing PSI-based orthognathic surgery with preoperative CBCT imaging during the study period were included. Use of PSI was an inclusion criterion. Patients imaged with conventional CT were excluded.

In addition to cleft patients, a small number of patients with other congenital craniofacial deformities were included because they required comparable VSP workflows and PSI-based reconstruction. Subgroup analyses were not performed due to limited sample size.

### CBCT imaging protocols and allocation

All scans were acquired using a Planmeca Viso G7 scanner (Planmeca Oy, Helsinki, Finland):

- FACE – standard-dose protocol (F2, measurement no. 3)
- JAW – ULD protocol (J13, measurement no. 8)

The operating surgeon referred the patient for imaging. The interpreting radiologist selected the CBCT protocol according to institutional practice and clinical assessment. No predefined allocation criteria were applied, and each patient underwent a single preoperative CBCT examination.

Field of view (FOV) was selected case-by-case based on anatomical and surgical requirements. Exposure parameters reflect routine clinical practice. Acquisition settings have been described previously [6]. The ULD protocol incorporates the Adaptive Image Noise Optimizer (AINO) [7].

### Study variables

#### Primary predictor

CBCT protocol (FACE vs JAW)

#### Primary outcomes

- Cutting guide fit
- PSI fit

Good fit was defined as stable seating without visible gaps or rocking and no need for intraoperative modification.

#### Secondary outcome

Occlusal outcome (as planned / compromise / reoperated).

#### Covariates

Age, sex, diagnosis, and type of orthognathic procedure.

### Data processing and STL generation

DICOM data were segmented using Planmeca Romexis^®^. STL models were generated by global thresholding. Virtual planning was performed using Geomagic Freeform 2021 (3D Systems, Rock Hill, SC, USA), and PSIs were manufactured from medically approved materials.

STL generation was performed by Planmeca ProModel. The workflow is standardized and compliant with medical device regulations. Segmentation and STL generation were conducted by experienced planning specialists (> 15 years of experience) following validated internal protocols to minimize operator-dependent variability. Surgeons did not manually modify STL files prior to manufacturing.

### Intraoperative assessment

All surgeries were performed by the same two experienced maxillofacial surgeons (JL, JS). Cutting guide fit and PSI fit were assessed intraoperatively using predefined clinical criteria and documented in operative reports. No postoperative imaging-based validation was performed in accordance with ALARA. Potential sources of deviation were categorized as imaging-related, technical, or surgical (Table 1).

**Table 1:** Qualitative categorization of potential sources of intraoperative fit deviations

Category	Description of issue	Potential contributing factor	Relation to image quality
Imaging-related	Increased image noise, reduced contrast	ULD protocol, small FOV	May affect segmentation accuracy
Technical (segmentation/STL)	Minor surface irregularities	Threshold selection, smoothing	Indirect
Surgical	Limited access, scar tissue tension	Intraoperative conditions	Not image-related

### Radiation dose assessment

Radiation exposure during the orthognathic surgical phase was quantified using DAP and effective dose.

### Statistical analysis

Continuous variables are presented as medians with IQRs. Normality was assessed using the Shapiro–Wilk test. Inter-group comparisons used independent-samples t-test or Mann–Whitney U test as appropriate. Cutting guide fit and PSI fit were analyzed using Fisher’s exact test.

Baseline differences between FACE and JAW groups were explored using bivariate analyses. Binary logistic regression was performed with CBCT protocol as the dependent variable and age, sex, diagnosis, and procedure type as independent variables. P-values < 0.05 were considered statistically significant.

## Results

### Patient characteristics

The study included 40 patients aged 12–34 years (median 17; IQR 14–22). Nine underwent surgery during growth (< 14 years). Diagnoses included unilateral cleft lip and palate (*n* = 23), bilateral cleft lip and palate (*n* = 10), and other craniofacial deformities (*n* = 7). Procedures comprised LF1 osteotomy (*n* = 32), bimaxillary osteotomy (*n* = 6), SRO (*n* = 1), and mandibular distraction with genioplasty (*n* = 1). Detailed characteristics are presented in Table 2, and individual patient-level data in Table 3.

**Table 2:** Patient characteristics of the study cohort. Age is presented as median with interquartile range and range. Surgical procedures and diagnoses are reported as absolute numbers and percentages

Variable	Value
Number of patients	40
Age (years), median (IQR, range)	17 (14–22, 12–34)
Sex, n (%)	
– Female	17 (42.5)
– Male	23 (57.5)
Diagnosis, n (%)	
– Unilateral cleft lip and palate (UCLP)	23 (57.5)
– Bilateral cleft lip and palate (BCLP)	10 (25.0)
– Non-cleft congenital craniofacial deformities	7 (17.5)
Surgical procedure, n (%)	
– Le Fort I osteotomy	32 (80.0)
– Bimaxillary osteotomy	6 (15.0)
– Mandibular sagittal split ramus osteotomy (SRO)	1 (2.5)
– Mandibular distraction osteogenesis with genioplasty	1 (2.5)
Surgery during growth (< 14 years), n (%)	9 (22.5)

**Table 3:** Individual patient characteristics and surgical details

Patient nr.	Sex	Age	Surgery type	Graft material	Cleft type	Diagnosis	Surgeon	Operation	Surgicalguide fit	Fit of plate	Final occlusion	Cleft palate fracture during oper.
1	F	16	Le Fort I	DBX	BLCP	Q37.0	JL, JS	Naive	good	good	as planned	no
2	M	12	Le Fort I	DBX	BLCP	Q37.0	JL	Naive	good	good	compromise	2 pieces
3	F	15	Le Fort I	DBX	BLCP	Q37.0	JL	Naive	good	good	as planned	no
4	F	16	Le Fort I	DBX	ULCP	Q37.1	JL, JS	reop.	good	problem	as planned	no
5	M	19	Le Fort I	DBX	ULCP	Q37.1	JL, JS	Naive	good	good	compromise	no
6	F	16	Le Fort I	DBX	ULCP	Q35.1	JL, JS	Naive	good	good	as planned	no
7	M	18	Le Fort I	DBX	ULCP	Q37.1	JL, JS	reop.	good	good	compromise	no
8	M	17	Le Fort I	DBX	ULCP	Q37.1	JS	Naive	good	good	as planned	no
9	F	16	Bimax	auto crista	none	Q75.1	JL, JS	Naive	good	good	as planned	no
10	M	13	Le Fort I	DBX	ULCP	Q35.1	JL, JS	Naive	good	good	as planned	no
11	F	25	Bimax	DBX	BLCP	Q37.1	JL, JS	Naive	good	good	as planned	no
12	M	16	Le Fort I	DBX	BLCP	Q37.0	JL, JS	Naive	good	good	as planned	3 pieces
13	M	18	Le Fort I	DBX	BLCP	Q37.0	JS	Naive	good	good	as planned	no
14	M	22	Le Fort I	DBX	none	Q67.3	JL, JS	Naive	good	good	as planned	no
15	F	30	Bimax	DBX	none	other	JL, JS	Naive	good	good	as planned	no
16	M	13	Le Fort I	DBX	BLCP	Q37.0	JL	Naive	good	good	as planned	no
17	M	17	Le Fort I	DBX	BLCP	Q37.0	JL	reop.	good	good	compromise	2 pieces
18	F	32	Le Fort I	DBX	ULCP	Q37.1	JL, JS	reop.	good	good	as planned	no
19	M	21	Le Fort I	DBX	none	other	JS	Naive	good	good	as planned	no
20	F	32	Le Fort I	DBX	ULCP	Q37.1	JL	Naive	good	good	as planned	no
21	M	14	Distract+genio	no graft	ULCP	other	JL, JS	Naive	good	good	as planned	no
22	F	16	Le Fort I	Grafton Putty	none	other	JL	Naive	good	good	as planned	no
23	M	12	Le Fort I	DBX	ULCP	Q37.1	JL	Naive	good	good	as planned	no
24	F	13	Le Fort I	Grafton Putty	ULCP	Q37.1	JL, JS	Naive	good	problem	compromise	no
25	F	12	Le Fort I	Grafton Putty	ULCP	Q37.1	JL, JS	Naive	good	good	as planned	bends
26	M	19	Bimax	auto crista + Grafton Putty	none	other	JS	Naive	good	good	reoperated	no
27	F	17	SRO	auto crista	none	other	JL, JS	Naive	good	good	as planned	no
28	M	12	Le Fort I	Grafton Putty	ULCP	Q37.1	JL	Naive	good	good	as planned	no
29	M	21	Le Fort I	auto crista + Grafton Putty	ULCP	Q37.1	JL, JS	Naive	good	problem	as planned	2 pieces
30	M	30	Le Fort I	Grafton Putty	ULCP	Q37.1	JL, JS	Naive	good	good	as planned	no
31	M	29	Le Fort I	Grafton Putty	ULCP	Q37.1	JL, JS	reop.	problem	good	as planned	no
32	M	13	Le Fort I	Grafton Putty	ULCP	Q37.1	JL, JS	Naive	good	good	as planned	no
33	M	13	Le Fort I	Grafton Putty	BLCP	Q37.0	JL, JS	Naive	good	good	as planned	bends
34	M	19	Le Fort I	Grafton Putty	ULCP	Q37.1	JL, JS	Naive	good	good	as planned	no
35	F	15	Bimax	Grafton Putty	ULCP	Q37.1	JL, JS	Naive	good	good	as planned	no
36	M	19	Le Fort I	Grafton Putty	ULCP	Q37.1	JL, JS	Naive	good	good	as planned	2 pieces
37	F	16	Le Fort I	Grafton Putty	BLCP	Q37.0	JL, JS	Naive	good	good	as planned	no
38	F	18	Bimax	Grafton Putty	ULCP	Q37.1	JL, JS	Naive	good	good	as planned	no
39	M	22	Le Fort I	Grafton Putty	ULCP	Q37.1	JL, JS	Naive	good	good	as planned	no
40	M	34	Le Fort I	Grafton Putty	ULCP	Q37.1	JL, JS	Naive	good	good	compromise	2 pieces

^®^Grafton Putty and DBX refer to commercially available demineralized bone matrix (DBM) products

*JS* Juho Suojanen, *JL* Junnu Leikola

**Fig. 1:**
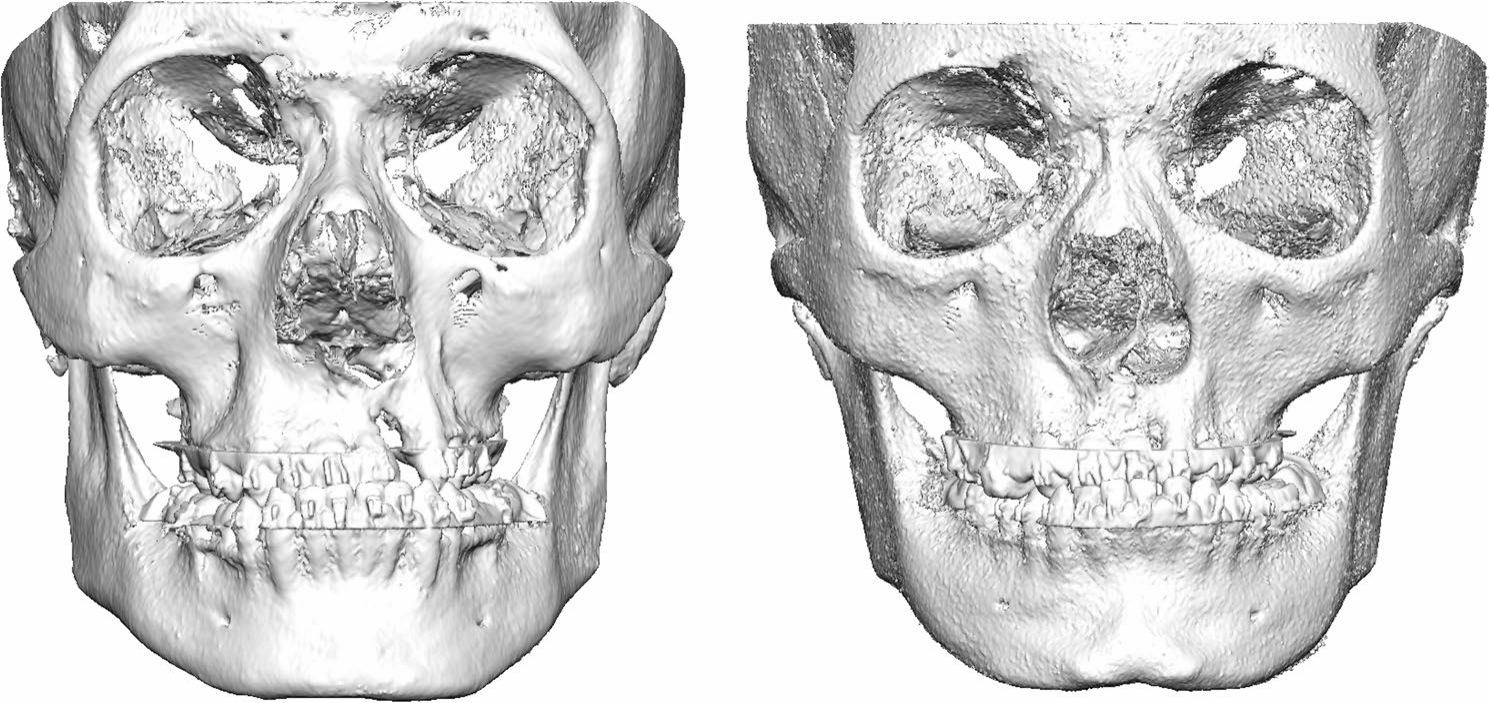
Preoperative frontal view before Le Fort I osteotomy. Left: CBCT image acquired using the standard-dose FACE protocol. Right: CBCT image acquired using the ULD JAW protocol

**Fig. 2:**
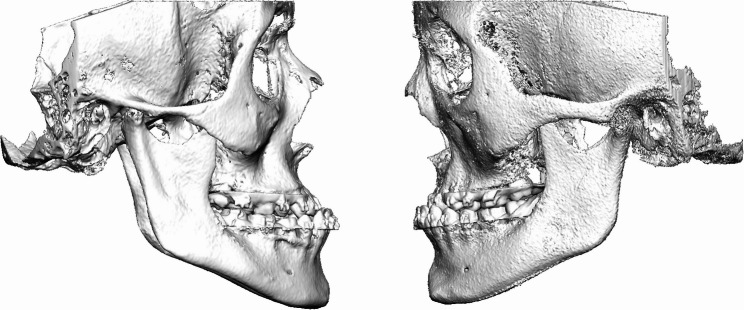
Preoperative situation, lateral view before Le Fort I osteotomy. Left: CBCT image acquired using the standard-dose FACE protocol. Right: CBCT image acquired using the ULD JAW protocol

**Fig. 3:**
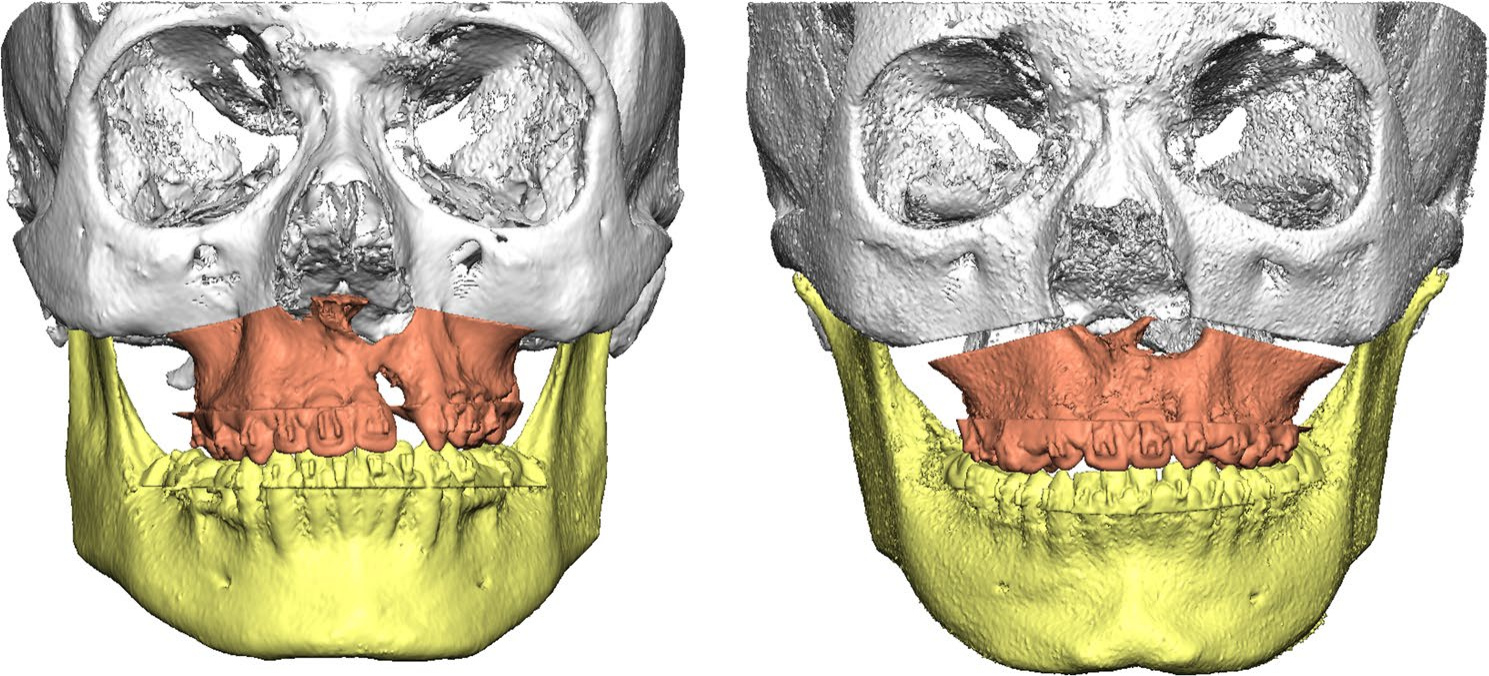
Postoperative situation, frontal view. Left: CBCT image acquired using the standard-dose FACE protocol. Right: CBCT image acquired using the ULD JAW protocol

**Fig. 4:**
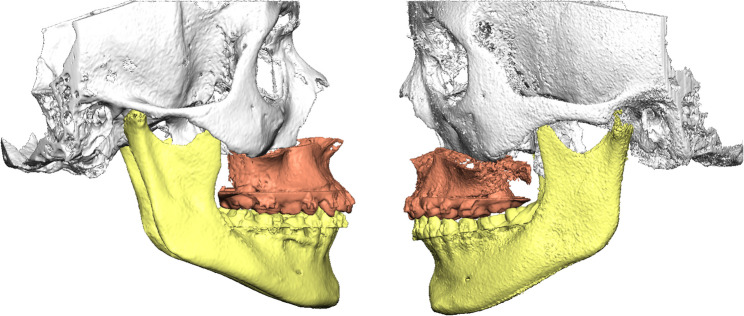
Postoperative situation, lateral view. Left: CBCT image acquired using the standard-dose FACE protocol. Right: CBCT image acquired using the ULD JAW protocol

Representative examples of preoperative and postoperative CBCT images obtained with the standard-dose (FACE) and ULD (JAW) protocols are shown in Figs. 1, 2, 3 and 4. These examples illustrate comparable anatomical visualization for virtual surgical planning.

### Radiation dose comparison

The JAW protocol resulted in significantly lower DAP and effective dose than the FACE protocol (effective dose 40.3 vs. 89.0 µSv; *p* < 0.001). No significant differences were observed in cutting guide fit (*p* = 0.450) or PSI fit (*p* = 0.238). Detailed exposure parameters are presented in Table 4.

**Table 4:** Exposure parameters

	Protocol		Dose	Voxel size	Tube voltage	Tube current	Exp. time	Q	FOV H x ø	†ED	DAP
Patient nr.	nr.	name	mode	[mm]	[kV]	[mA]	[s]	[mAs]	[cm]	[µSv]	[mGy⋅ cm^2^]
1	3	FACE	Normal	0.3	90	14	4.5	63.0	16 × 16	89	641
2	3	FACE	Normal	0.3	90	14	4.5	63.0	16 × 16	89	641
3	3	FACE	Normal	0.3	90	14	4.5	63.0	14 × 14	89	515
4	3	FACE	Normal	0.3	90	14	4.5	63.0	16 × 16	89	641
5	3	FACE	Normal	0.3	90	14	4.5	63.0	16 × 16	89	641
6	3	FACE	Normal	0.3	90	14	4.5	63.0	16 × 16	89	641
7	3	FACE	Normal	0.3	90	14	4.5	63.0	16 × 16	89	641
8	8	JAW	ULD	0.15	100	6	2.5	15.2	16 × 16	43	344
9	3	FACE	Normal	0.3	90	14	4.5	63.0	15 × 16	83	613
10	†8	JAW	†ULD	0.15	90	8	2.5	20.0	12 × 16	29	238
11	8	JAW	ULD	0.15	100	6	2.5	15.2	14 × 16	38	306
12	8	JAW	ULD	0.15	100	6	2.5	15.2	15 × 16	40	325
13	8	JAW	ULD	0.15	100	6	2.5	15.2	14 × 16	38	306
14	8	JAW	ULD	0.15	100	6	2.5	15.2	14 × 15	35	292
15	8	JAW	ULD	0.15	100	6	2.5	15.2	15 × 16	40	325
16	3	FACE	Normal	0.3	90	14	4.5	63.0	16 × 16	89	650
17	3	FACE	Normal	0.3	90	14	4.5	63.0	18 × 18	113	636
18	8	JAW	ULD	0.15	100	6	2.5	15.2	15 × 16	40	325
19	3	FACE	Normal	0.3	90	14	4.5	63.0	18 × 18	113	668
20	8	JAW	ULD	0.15	100	6	2.5	15.2	16 × 16	43	275
21	3	FACE	Normal	0.3	90	14	4.5	63.0	13 × 16	72	532
22	3	FACE	Normal	0.3	90	14	4.5	63.0	13 × 16	72	519
23	3	FACE	Normal	0.3	90	14	4.5	63.0	16 × 16	89	519
24	3	FACE	Normal	0.3	90	14	4.5	63.0	16 × 16	89	550
25	3	FACE	Normal	0.3	90	14	4.5	63.0	14 × 14	68	438
26	3	FACE	Normal	0.3	90	14	4.5	63.0	19 × 18	119	700
27	8	JAW	ULD	0.15	100	6	2.5	15.2	16 × 16	43	241
28	8	JAW	ULD	0.15	100	6	2.5	15.2	16 × 16	43	258
29	3	FACE	Normal	0.3	90	14	4.5	63.0	16 × 16	89	550
30	8	JAW	ULD	0.15	100	6	2.5	15.2	16 × 16	43	291
31	8	JAW	ULD	0.15	100	6	2.5	15.2	16 × 16	43	344
32	3	FACE	Normal	0.3	90	14	4.5	63.0	17 × 16	95	581
33	†3	FACE	†Normal	0.3	100	12	4.0	48.0	14 × 14	74	678
34	3	FACE	Normal	0.3	90	14	4.5	63.0	16 × 16	89	551
35	8	JAW	ULD	0.15	100	6	2.5	15.2	14 × 16	38	259
36	8	JAW	ULD	0.15	100	6	2.5	15.2	15 × 15	38	263
37	8	JAW	ULD	0.15	100	6	2.5	15.2	15 × 16	40	275
38	‡8	JAW	‡ULD	0.15	100	10	2.5	25.0	14 × 15	58	387
39	3	FACE	Normal	0.3	90	14	4.5	63.0	18 × 22	138	784
40	8	JAW	ULD	0.15	100	6	2.5	15.2	16 × 16	43	292

†ED is effective dose

### Baseline comparison between protocol groups

In logistic regression analysis, age was significantly associated with CBCT protocol allocation (*p* = 0.014). Sex (*p* = 0.45), procedure type (*p* = 1.00), and diagnosis (*p* = 0.894) were not significantly associated. Apart from age, baseline characteristics were comparable between groups.

### Surgical outcomes

PSI fit was clinically acceptable in 37 patients (92.5%). One cutting guide misfit (2.5%) was corrected intraoperatively. Three PSI misfits (7.5%) occurred due to screw hole misalignment or seating limitations. One patient required reoperation for postoperative malocclusion. No statistically significant differences in intraoperative outcomes were observed between protocols.

## Discussion

This study evaluated the clinical applicability of standard-dose and ULD CBCT protocols in PSI-based orthognathic surgery for patients with cleft lip and palate and other congenital craniofacial deformities. These patients frequently undergo repeated imaging during growth and adolescence, making cumulative radiation exposure a relevant long-term concern. Within this context, the present findings demonstrate that the ULD protocol achieved a substantial reduction in radiation dose while maintaining intraoperative cutting guide and PSI fit comparable to the standard-dose protocol.

From a reconstructive and orthognathic surgical standpoint, the primary concern is whether reduced-dose imaging compromises the reliability of virtual planning and implant manufacturing. Cutting guide and PSI fit are clinically meaningful endpoints because they reflect the entire digital workflow—from image acquisition and segmentation to STL model generation, implant design, and intraoperative application. Comparable intraoperative performance between protocols suggests that dose reduction did not impair the practical accuracy required for PSI-based maxillary repositioning in complex cleft-related anatomy.

Importantly, implant misfit cannot be attributed solely to imaging quality. Technical factors such as segmentation thresholds, STL smoothing, and screw trajectory planning may influence final adaptation. In addition, intraoperative conditions—including scar-related palatal tension, asymmetry, limited exposure, or mechanical resistance during repositioning—may affect seating. Thus, PSI fit represents an integrated outcome rather than a pure measure of image resolution. The absence of significant differences between protocols indicates that ULD CBCT provides sufficient structural information for clinically reliable execution.

Although conventional CT remains the reference standard in complex craniofacial reconstruction [8], optimized CBCT protocols have demonstrated adequate volumetric accuracy for STL-based planning while offering lower radiation exposure [6]. PSI-based workflows have been successfully implemented in cleft populations, where anatomical variability and prior surgical scarring increase procedural complexity [9]. Surface-based planning processes are known to be sensitive to segmentation and registration inaccuracies [10], emphasizing the importance of standardized STL generation procedures. Previous investigations have supported the feasibility of low-dose and ULD CBCT protocols in maxillofacial and implant-related applications [7, 11–15], and the present results extend these findings to PSI-based orthognathic surgery in a pediatric-onset craniofacial cohort.

Age was associated with protocol allocation in this study. This likely reflects clinical preference for radiation minimization in younger patients rather than systematic selection bias, as other demographic and procedural variables were comparable between groups. This observation underscores the real-world applicability of dose optimization strategies in growing patients.

Several limitations should be acknowledged. The cohort size was modest and derived from a single tertiary referral center using one CBCT device, which may limit generalizability. Intraoperative assessment of PSI fit was qualitative and based on routine surgical documentation. Quantitative postoperative deviation analysis was not performed to avoid additional radiation exposure in accordance with the ALARA principle. Furthermore, field-of-view selection was not standardized and may have influenced dose measurements. Nevertheless, the study reflects real-world clinical conditions in cleft and craniofacial surgery.

Taken together, the findings suggest that ULD CBCT can be integrated into complex orthognathic workflows without compromising surgical predictability. In patients who require repeated imaging throughout growth, such dose reduction strategies may meaningfully decrease cumulative exposure while preserving operative reliability.

## Conclusion

Both FACE (standard-dose) and JAW (ULD) CBCT protocols provided image data sufficient for virtual surgical planning and PSI-based orthognathic surgery in patients with cleft and other congenital craniofacial deformities. No significant differences were observed in intraoperative cutting guide or PSI fit between protocols.

While the ULD protocol achieved substantial radiation reduction, the results indicate that both imaging approaches are clinically reliable within a PSI-based digital workflow. In patients requiring repeated imaging during growth, dose optimization through ULD CBCT may reduce cumulative radiation exposure without compromising surgical accuracy. Further prospective studies with quantitative postoperative validation are warranted.

## Data Availability

The datasets generated and/or analyzed during the current study are available from the corresponding author on reasonable request.

## Abbreviations

3D: Three—dimensional
AINO: Adaptive Image Noise Optimizer
ALARA: As Low As Reasonably Achievable
CAD/CAM: Computer—Aided Design/Computer—Aided Manufacturing
CBCT: Cone—Beam Computed Tomography
CNR: Contrast—to—Noise Ratio
CT: Computed Tomography
DAP: Dose—Area Product
DBX: Demineralized Bone Matrix
HR: High Resolution
IQR: Interquartile Range
LF1: Le Fort I Osteotomy
MMF: Maxillomandibular Fixation
MTF: Modulation Transfer Function
PSI: Patient—Specific Implant
SNR: Signal—to—Noise Ratio
SRO: Sagittal Split Ramus Osteotomy
STL: Standard Tessellation Language (3D model file format)
ULD: Ultra—Low Dose
VSP: Virtual Surgical Planning
